# Metagenome Sequencing of Prokaryotic Microbiota from Two Hypersaline Ponds of a Marine Saltern in Santa Pola, Spain

**DOI:** 10.1128/genomeA.00933-13

**Published:** 2013-11-14

**Authors:** Ana B. Fernandez, Rohit Ghai, Ana Belen Martin-Cuadrado, Cristina Sanchez-Porro, Francisco Rodriguez-Valera, Antonio Ventosa

**Affiliations:** Department of Microbiology and Parasitology, Faculty of Pharmacy, University of Seville, Seville, Spaina; Evolutionary Genomics Group, División de Microbiología, Universidad Miguel Hernández, San Juan, Alicante, Spainb

## Abstract

Marine salterns are composed of several shallow ponds with a salinity gradient, from seawater to salt saturation, with gradually changing microbial populations. Here, we report the metagenome sequencing of the prokaryotic microbiota of two ponds with 13% and 33% salinity from a saltern in Santa Pola, Spain.

## GENOME ANNOUNCEMENT

Hypersaline environments are widely distributed habitats characterized by their high salt concentrations, e.g., solar salterns, saline soils, salted foods, and other salted products (1). Among these, salterns are probably the most widely studied habitats and are excellent models for studying the microbial populations inhabiting the ponds along a natural salinity gradient up to NaCl saturation (2, 3). One of the more extensively studied salterns is located in Santa Pola, near Alicante in southeastern Spain. These salterns have been the subjects of a variety of studies, with approaches ranging from the classical culture-dependent to the more recent culture-independent methods, over nearly 30 years (4–10). A recent metagenomic study described the microbiota of two of these saltern ponds, one of intermediate salinity (19% salt) and an NaCl-saturated crystallizer pond (37% salt), using direct pyrosequencing (11).

This study is aimed toward investigating the prokaryotic microbiota of the surface water of two additional hypersaline ponds, with 13% and 33% salinity, in the same location (Bras del Port saltern, Santa Pola, Spain; GPS coordinates: for the 13% pond [SS13], 38°12′05″N, 0°35′44″W; and for the 33% pond [SS33], 38°11′59″N, 0°35′11″W). Fifty-liter water samples were collected on 20 September 2010 and sequentially filtered through 5.0-µm and 0.22-µm pore polycarbonate filters (Millipore, USA). Metagenomic DNA was extracted and purified as previously described (11, 12). The purified total prokaryotic DNA was sequenced by pyrosequencing (Roche 454 GS-FLX system, Titanium chemistry) by GATC, Konstanz, Germany. The average read lengths obtained were 305 bp and 367 bp for the SS13 and SS33 metagenomes, respectively. A total of 1,443,593 reads and 842,872 reads were obtained for SS13 and SS33, respectively.

The sequences were compared against the Ribosomal Database Project (RDP) (13) to analyze and identify 16S rRNA genes. Metagenomic reads were also annotated using the MG-RAST server (14), CAMERA (15), and IMG (16).

The most abundant phylum detected in both samples was *Euryarchaeota*, while the phyla *Bacteroidetes* and *Euryarchaeota* were the only two prokaryotic groups shared by the metagenomic datasets. At 13% salinity, the diversity found at the phylum and genus level was higher than in the 33% salinity saltern, but the number of sequences unclassified at the genus level was higher in the 13% salinity saltern.

The data from these metagenomic datasets reveal the genetic diversity in these ponds and the changes in microbial community composition along the salinity gradient in this environment compared to the metagenomic data available from different ponds from this saltern.

### Nucleotide sequence accession number.

The DNA sequences from this metagenomic project have been deposited in the NCBI Sequence Read Archive under the accession no. SRP028290.
